# Clinical and Biochemical Features of Hypopituitarism Among Brazilian Children With Zika Virus–Induced Microcephaly

**DOI:** 10.1001/jamanetworkopen.2021.9878

**Published:** 2021-05-13

**Authors:** Leda L. Ferreira, Juan P. Aguilar Ticona, Paulo S. Silveira-Mattos, María B. Arriaga, Thaisa B. Moscato, Gildásio C. Conceição, Antonio Carlos dos Santos, Federico Costa, Crésio A.D. Alves, Sonir R. Antonini

**Affiliations:** 1 Programa de Pós-graduação em Medicina e Saúde Humana, Escola Bahiana de Medicina e Saúde Pública, Salvador, Bahia, Brazil; 2 Hospital University Hospital Professor Edgard Santos, Universidade Federal da Bahia, Salvador, Bahia, Brazil; 3 Instituto Gonçalo Moniz, Fundação Oswaldo Cruz, Salvador, Bahia, Brazil; 4 Instituto de Saúde Coletiva, Universidade Federal da Bahia, Salvador, Bahia, Brazil; 5 Faculdade de Medicina da Bahia, Universidade Federal da Bahia, Bahia, Brazil; 6 Associação de Pais e Amigos dos Excepcionais, Salvador, Bahia, Brazil; 7 Department of Medical Imaging, Hematology and Oncology, Ribeirao Preto Medical School, University of Sao Paulo, Sao Paulo, Brazil; 8 Department of Pediatrics, Ribeirao Preto Medical School, University of Sao Paulo, Sao Paulo, Brazil

## Abstract

**Importance:**

The Zika virus infects progenitor neuron cells, disrupts cerebral development, and, in mice, drives hypothalamic defects. Patients with microcephaly caused by congenital Zika infection present with midline cerebral defects, which may result in hypopituitarism.

**Objective:**

To analyze postnatal growth and the presence of clinical and biochemical features associated with hypopituitarism in children with congenital Zika infections.

**Design, Setting, and Participants:**

In this prospective cohort study at 2 public referral hospitals in Bahia, Brazil, specializing in the treatment of congenital Zika infection, clinical data and growth parameters of 65 patients with the infection were evaluated. Data were analyzed from April 2017 through July 2018.

**Exposure:**

Congenital Zika infection.

**Main Outcomes and Measures:**

Length, weight, and head circumference were measured at birth and during follow up (ie, at 27 months of life) for each patient. Basal levels of free thyroxine, thyrotropin, cortisol, corticotropin, prolactin, insulin-like growth factor 1, insulin-like growth factor binding protein 3, urine and plasma osmolality, electrolytes, glucose, and insulin were evaluated at the age of 26 months to 28 months. All patients underwent central nervous system computed tomography scans and ophthalmic and otoacoustic evaluations at the time of this investigation or had done so previously.

**Results:**

Among 65 patients (38 [58.4%] male; median [interquartile range] age at enrollment, 27 [26-28] months), 61 patients presented with severe brain defects (93.8%), including corpus callosum agenesis or hypoplasia (ie, midline brain defects; 25 patients [38.5%]) and optic nerve atrophy (38 patients [58.5%]). Most patients presented with severe neurodevelopmental delay (62 of 64 patients [96.9%]). Past or present clinical signs of hypopituitarism were rare, occurring in 3 patients (4.6%). Severe microcephaly, compared with mild or moderate microcephaly, was associated with a shorter length by median (interquartile range) *z* score at birth (−1.9 [−2.5 to −1.0] vs −0.3 [−1.0 to 0]; *P* < .001), but this difference did not persist at 27 months (−1.6 [−2.3 to −0.3] vs −2.9 [−4.0 to −1.2]; *P* = .06). Growth hormone deficiency or hypothyroidism were not observed in any patients, and glucose and insulin levels were within reference ranges for all patients. Low cortisol levels (ie, below 3.9 µg/dL) were observed in 4 patients (6.2%). These 4 patients presented with low (ie, below 7.2 pg/mL) or inappropriately low (ie, below 30 pg/mL) corticotropin levels. Low corticotropin levels (ie, below 7.2 pg/mL) were observed in 6 patients (9.2%). Diabetes insipidus was evaluated in 21 patients; it was confirmed in 1 patient (4.8%) and suggested in 3 patients (14.3%).

**Conclusions and Relevance:**

This study found that congenital Zika infection with microcephaly was associated with midline brain defects and optic nerve atrophy. Children with congenital Zika infections presented with prenatal growth impairments with a lack of postnatal catch-up, as shown by persistent short length from birth until 27 months; these impairments were not associated with growth hormone deficiency. Patients also presented with severe developmental delay that was not associated with hypothyroidism, while central adrenal insufficiency and diabetes insipidus occurred in some patients.

## Key Points

**Question:**

Do patients with microcephaly and central nervous system malformations caused by congenital Zika virus infection present with hormone deficiencies?

**Findings:**

In this cohort study of 65 patients with congenital Zika virus infection, most patients presented with severe brain defects. Severe developmental delay and prenatal growth impairment with no postnatal catch-up growth occurred frequently, but they were not associated with growth hormone deficiency or hypothyroidism, and few of these patients presented with central adrenal insufficiency or diabetes insipidus in the third year of life.

**Meaning:**

These findings suggest that hypopituitarism is infrequent within the first years of life in children with congenital Zika infections.

## Teaser

This cohort study investigates postnatal growth and hormone levels among Brazilian children with microcephaly caused by Zika virus.

## Introduction

Some emerging viral infections resulting in epidemics or pandemics can have clinical impacts on patients, not only during the infection period, but also throughout their lives. The consequences of these infections can include lifelong health issues, as observed in the offspring of pregnant women infected with Zika virus.

Zika virus was first isolated in 1947 in a forest called Zika in Uganda, Africa, where the first confirmed human infection cases were reported in 1962.^1^ The first Zika virus epidemics occurred in the Pacific region, specifically in Micronesia, from 2007 to 2013. The virus was introduced to northeast Brazil between 2013 and 2015 before spreading throughout the Americas.^2^ One of the epicenters of the Zika virus epidemic in northeast Brazil was Bahia. As a result of this epidemic, there was a massive increase in the incidence of congenital microcephaly. From 2015 through 2016, congenital Zika virus syndrome (CZS) caused approximately 83% of the microcephaly cases in Brazil.^3^

This syndrome comprises a wide range of congenital defects resulting from the vertical transmission of Zika virus infections during gestation. Neurotrophic Zika virus infects progenitor neuron cells, increases neuronal apoptosis, and deregulates the cell cycle, resulting in impaired neuronal migration and brain development.^4^ In this context, the most common defects resulting from CZS are microcephaly, joint contractures, global neurodevelopmental delay, epilepsy, visual impairment, and deafness.^4,5^ Severe congenital microcephaly is the most severe manifestation of CZS.^4^

Midline brain defects, such as optic nerve hypoplasia and corpus callosum and septum pellucidum agenesis have been reported in patients with CZS. Septo-optic dysplasia comprises at least 2 of the following findings: optic nerve hypoplasia, corpus callosum or septum pellucidum agenesis, and hypopituitarism.^6^ The association between midline brain defects and hypothalamic-pituitary hormone deficiencies (isolated or combined hypopituitarism) is well known and results in deficient growth and pubertal development, reduced bone mass and fertility, and decreased quality of life.^6,7,8,9,10,11^ To date, there is limited information on hypothalamic and pituitary function in patients with CZS;^12^ however, hypothalamic defects have been observed in an experimental Zika virus mouse model. These findings suggest the possibility of multiple hypothalamic-pituitary hormone deficiencies and growth impairments in children with CZS.^13^ Therefore, we hypothesized that children with severe central nervous system malformations caused by CZS would present hormone pituitary deficiencies. In this study, we evaluated the clinical and biochemical signs associated with hypothalamic-pituitary hormone deficiencies in toddlers with microcephaly associated with CZS.

## Methods

This cohort study was conducted according to the guidelines set by the Declaration of Helsinki, and the ethical committees of the Hospital Geral Roberto Santos and University Hospital Professor Edgard Santos approved the study (No. 1.886.918). The patients’ parents or legal caregivers provided written informed consent. The race and ethnicity of the patients were not assessed.

### Patients

#### Study Design, Participant Eligibility, and Data Collection

This prospective cohort study was conducted from January 2015 through December 2018. We evaluated children with Zika virus–associated microcephaly who were followed up at Hospital Geral Roberto Santos and University Hospital Professor Edgard Santos in the city of Salvador in Bahia, Brazil; both institutions are referral public hospitals for pediatric infectious diseases. We included all eligible infants with microcephaly epidemiologically associated with CZS who were born from January 2015 to April 2016. A finding of a head circumference of more than 2 SDs below the mean using International Fetal and Newborn Growth Consortium for the 21st Century (INTERGROWTH-21st) curves^14^ confirmed the diagnosis of microcephaly. Children with head circumferences more than 3 SDs below the mean at the time of the study, when they were median (interquartile range [IQR]) age 27 (26-28) months, were classified as having severe microcephaly.^15^ We classified congenital microcephaly as being associated with Zika virus infection after excluding other common congenital infections and conditions associated with microcephaly. All patients included in this study exhibited undetectable IgM and IgG antibodies against toxoplasmosis, rubella, and cytomegalovirus (using enzyme-linked immunoassay) or syphilis (using venereal disease research laboratory test). The presence of microcephaly and typical central nervous system (CNS) defects were documented by brain computed tomography (CT) scans and transfontanelle ultrasonography in the epidemiological context of Zika epidemic–defined CZS. The CT findings were extracted from existing radiology reports. Additional clinical features considered in the diagnosis of CZS included other defects commonly found among these patients, such as retinal alterations, hypertonia, and muscle contractures. Therefore, the diagnosis of CZS was primarily epidemiological for children born in an epidemic region during the Zika virus outbreak. This diagnosis was strengthened by the specific findings from CT scans and eye defects that are most specific to CZS.

All patients were evaluated by a multidisciplinary team (consisting of a pediatric neurologist, a pediatric ophthalmologist, a physiotherapist, a speech therapist, and nurses) at least every 3 months after birth. Clinical evaluations, performed by a pediatric neurologist and a pediatrician specializing in infectious diseases (L.L.F.), included measurement of length or height, weight, and head circumference of each patient. Experienced pediatric neurologists performed clinical evaluations to assess the presence or absence of developmental milestones, hypertonia, the persistence of primitive reflexes, spasticity, spasms, and postural defects. The presence of swallowing and sucking problems was also evaluated. Patients underwent ophthalmic examinations, including retinal mapping, fundoscopy, optic nerve evaluations, otoacoustic emissions evaluations, and brain-evoked response audiometry evaluations.

We assessed past and present clinical signs of anterior hypopituitarism, including micropenis and cryptorchidism, delayed umbilical cord separation, poor sucking, large wide-open fontanelles, macroglossia, prolonged neonatal jaundice, neonatal hypoglycemia, and poor weight and length gain. We also assessed clinical signs associated with diabetes insipidus, such as polyuria, hyperthermia, and hypernatremia. Glucose, sodium, and potassium levels were analyzed using an autoanalyzer. Morning (ie, 8-9 am) basal levels of free thyroxine (FT_4_), thyrotropin (formerly thyroid-stimulating hormone), cortisol, corticotropin (formerly adrenocorticotropic hormone), prolactin, insulin-like growth factor 1 (IGF-1), and insulin-like growth factor binding protein 3 (IGFBP3) were measured in a single reference laboratory using an immunochemiluminometric assay (at the Laboratory of the Associação de Pais e Amigos dos Excepcionais). Plasma osmolality and urine osmolality were measured using a freezing point depression osmometer (Advanced Instruments) at the Laboratory of Endocrinology of the University Hospital at Ribeirao Preto Medical School, University of Sao Paulo.

### Statistical Analysis

Patients with CZS were analyzed comparing 3 outcomes: according to the presence of severe and nonsevere microcephaly, according to the presence or absence of corpus callosum defects, and comparing the presence or absence of retinal defects. Mild to moderate microcephaly was defined as having a head circumference from 2 to 3 SDs below the mean, and severe microcephaly was defined as having a head circumference more than 3 SDs below the mean.

Categorical variables were summarized as frequencies and percentages, and continuous variables were described as medians and interquartile ranges. The Mann-Whitney U test (for continuous variables), Fisher exact test (2 × 2 comparisons), or Pearson χ^2^ test (for categorical variables) were used to evaluate differences between groups. Hypothesis tests were 2-sided. Differences were considered statistically significant at *P* < .05. All analyses were performed using R statistical software version 3.1.6. (R Project for Statistical Computing). Data were analyzed from October 2019 through September 2020.

## Results

We evaluated 65 patients (38 [58.4%] male) with microcephaly due to CZS. Among 60 patients with data on preterm birth, 11 patients (18.3%) were preterm. (The sample size variation in some features reflects the availability of specific information.) The median (IQR) age at enrollment was 27 (26-28) months (Table 1). At birth, 36 patients (55.4%) presented with severe microcephaly and 29 patients (44.6%) presented with mild to moderate microcephaly. Overt clinical signs of Zika virus infection during gestation were present in 54 mothers (83.1%). The occurrence of symptoms in the first trimester was significantly higher in mothers of children with severe microcephaly than in mothers of children with nonsevere microcephaly (26 of 30 mothers [86.7%] vs 13 of 24 mothers [54.2%]; *P* = .02) (eTable 1 in the Supplement). Upon CT scan examination, all patients presented with CNS defects (eTable 2 in the Supplement), including some features most specific to congenital Zika infections; among 48 patients with calcification in CT scan images, this included 21 patients with subcortical calcifications (43.8%), 20 patients with periventricular calcifications (41.7%), 14 patients with basal nuclei calcifications (29.2%), and 14 patients with all of these features. In addition, among 33 patients who presented ophthalmological alterations, typical ophthalmological alterations were observed, including macular scarring in 16 patients (48.5%), mottled pigmentary retinopathy in 9 patients (27.3%), optic nerve defects in 6 patients (18.2%]), and 2 or more of these features in 10 patients (30.3%). Patients with severe microcephaly also presented with congenital contractures or arthrogryposis, retinal defects, and hearing loss (Table 1). Most patients presented with severe impairment of mobility; congenital contractures were observed in 18 of 64 patients (28.1%), and 62 of 64 patients (96.9%) presented with gross motor developmental delay.

**Table 1.** Patient Clinical and Developmental Characteristics by Degree of Microcephaly

Characteristic^a^	With severe microcephaly (n = 36)		With mild or moderate microcephaly (n = 29)		*P* value
	Sample size, No.	No. (%)	Sample size, No.	No. (%)	
Sex					
Male	36	22 (61.1)	29	16 (55.2)	.63
Female	36	14 (38.9)	29	13 (44.8)	
Preterm	36	7 (19.4)	24	4 (16.7)	.59
At birth					
Brain defect	36	36 (100)	29	29 (100)	NA
Head circumference *z*-score, median (IQR)	36	−4.3 (−4.8 to −3.9)	29	−2.4 (−2.8 to −2.1)	<.001
Length *z* score, median (IQR)^a^	36	−1.9 (−2.5 to −1.0)	24	−0.3 (−1.0 to 0.0)	<.001
Length *z* score^a^					
>SDs below mean	33	20 (60.6)	21	5 (23.8)	.02
≤2 SDs below mean	33	13 (39.4)	21	16 (76.2)	
Weight *z* score, median (IQR)^a^	33	−2.6 (−3.1 to −1.4)	21	−1.2 (−1.9 to −0.3)	.001
Weight *z* score^a^					
>2 SDs below mean	36	16 (44.4)	24	1 (4.2)	.002
≤2 SDs below mean	36	20 (55.6)	24	23 (95.8)	
Congenital contracture	35	14 (40.0)	29	4 (13.8)	.04
Neonatal seizure	35	6 (17.1)	29	2 (6.9)	.40
Ophthalmological alteration					
Retinal anomaly	35	28 (80.0)	27	10 (37.0)	<.001
Macular scarring	24	9 (37.5)	9	7 (77.8)	.10
Mottled pigmentary retinopathy	24	7 (29.2)	9	2 (22.2)	>.99
Optic nerve defect	24	5 (20.8)	9	1 (11.1)	.89
More than 1 defect	24	9 (37.5)	9	1 (11.1)	.30
At 27 mo					
Head circumference *z* score, median (IQR)^b^	23	−7.0 (−8.2 to −4.7)	25	−4.9 (−6.0 to −3.4)	.006
Weight *z* score, median (IQR)^b^	25	−1.1 (−3.2 to 0.3)	25	−1.8 (−2.4 to −0.3)	.97
Weight *z* score^b^					
>3 SDs below mean	25	8 (32.0)	25	3 (12.0)	.18
2 SDs to 3 SDs below mean	25	3 (12.0)	25	6 (24.0)	
<2 SDs below mean	25	14 (56.0)	25	16 (64.0)	
Length *z* score, median (IQR)^b^	25	−2.9 (−4.0 to −1.2)	25	−1.6 (−2.3 to −0.3)	.06
Length *z* score^b^					
>3 SDs below mean	25	12 (48.0)	25	4 (16.0)	.05
2 SDs to 3 SDs below mean	25	2 (8.0)	25	4 (16.0)	
<2 SDs below mean	25	11 (44.0)	25	17 (68.0)	
Head circumference *z* score^c^					
>3 SDs below mean	23	21 (91.3)	25	22 (88.0)	>.99
2 SDs to 3 SDs below mean	23	2 (8.7)	25	3 (12.0)	
Hearing loss	29	10 ( 34.5)	25	2 (8.0)	.04
Seizure	36	29 (80.5)	29	18 (62.1)	.10
Swallowing disorder	35	12 ( 34.3)	28	7 (25.0)	.42
Poor weight gain	35	15 ( 42.9)	28	12 (42.9)	>.99
Sleep disorder	36	20 (55.5)	29	13 ( 44.8)	.39
Severe language delay	35	34 (97.1)	29	27 ( 93.1)	.86
Severe fine motor delay	35	34 (97.1)	29	28 ( 96.6)	>.99
Severe gross motor delay	35	34 (97.1)	29	28 (96.6)	>.99

Abbreviations: IQR, interquartile range; NA, not applicable.

^a^ Data were compared using Fisher exact test. The total number of patients in each group was 36 and 29, respectively. The sample size variation in some features reflects the availability of specific information.

^b^ International Fetal and Newborn Growth Consortium for the 21st Century (INTERGROWTH-21st) parameters.

^c^ World Health Organization parameters.

Among all patients, past or present clinical signs associated with hypopituitarism were infrequent, occurring in 3 patients (4.6%). No patients presented with neonatal hypoglycemia or jaundice. Seizures occurred in 8 patients in the neonatal period (12.3%) and 47 patients at 27 months (72.3%), and 2 patients (3.1%) had persistent wide-open fontanelles. Cryptorchidism, but not micropenis, was observed in 1 patient (1.5%). This patient also presented with arthrogryposis.

Table 2 and eTable 2 in the Supplement show the results of basal hormone measurements. According to the assays’ reference values adjusted for age, none of the patients presented with central or primary hypothyroidism, as the FT_4_ and thyrotropin levels were within reference ranges for all patients. Glucose and insulin levels were also within reference ranges for all patients. For most patients (49 patients [75.4%]) in this study, basal cortisol levels ranged from 5 and to 13 μg/dL (to convert to nanomoles per liter, multiply by 27.588). Basal cortisol and corticotropin levels did not differ between patients with severe and nonsevere microcephaly. Low corticotropin levels (ie, below 7.2 pg/mL; to convert to picomoles per liter, multiply by 0.22) were observed in 6 patients (9.2%). Low cortisol levels (ie, below 3.9 µg/dL) were observed in 4 patients (6.2%). These 4 patients presented low (ie, below 7.2 pg/mL) or inappropriately low (ie, below 30 pg/mL) corticotropin levels. Among all patients, 9 patients (13.8%) presented with basal morning cortisol levels of less than 5 µg/dL; 1 of these patients had a basal morning cortisol level of less than 3 µg/dL indicating central adrenal insufficiency, and 8 of these patients had levels of from 3 to 5 µg/dL. The patient with a cortisol level less than 3 µg/dL presented with corticotropin level in the reference range (25.4 pg/mL) and a glucose level in the reference range (80 mg/dL; to convert to millimoles per liter, multiply by 0.0555). There were 4 patients (6.2%) with cortisol levels below 3.9 µg/dL. Among those patients with cortisol levels ranging from 3 to 5 µg/dL, 2 patients also had low corticotropin levels (ie, <7 pg/mL). None of these patients had a known history of present or recent synthetic corticosteroid use. Cortisol stimulation tests were not performed for these patients because their caregivers refused further investigation at the time of the study. However, these caregivers received counseling on the risk of acute adrenal insufficiency, especially during illness episodes.

**Table 2.** Patient Hormonal and Biochemical Profiles by Degree of Microcephaly

Characteristic^a^	With severe microcephaly (n = 36)		With mild or moderate microcephaly (n = 29)		*P* value
	Sample size, No.	Median (IQR)	Sample size, No.	Median (IQR)	
Age, mo	36	27 (25-29)	29	27 (25-28)	.66
Free thyroxine (RV: 0.54-1.43), ng/dL	35	0.9 (0.8-1)	29	0.9 (0.8-1.1)	.76
Thyrotropin (RV: 0.5-6.6), mIU/L	35	2.3 (1.6-3.5)	29	2.6 (2.1-3.9)	.26
Cortisol (RV: 5-18), μg/dL	36	7.5 (6.7-9.7)	29	8.3 (6.9-10.6)	.30
Corticotropin (RV: 7- 30), pg/mL	35	14 (9-20)	29	15 (11-19)	.72
IGF-1 (RV: 49-270), ng/mL	35	162 (118-226)	29	129 (91-163)	.01
IGFBP-3 (RV: 0.8-3.9), μg/mL	35	3.4 (2.6-4.2)	29	3.1 (1.9-3.5)	.10
Prolactin (RV: 2.6-25), ng/mL	35	7.4 (4.4–12)	29	8.4 (5.8–12)	.48
Glucose (RV: 70-100), mg/dL	31	82 (76-87)	29	79 (74-88)	.58
Insulin (RV: 2.5-25), mIU/L	35	7.2 (4.6-15)	29	4 (2-8.3)	.01
Urea (RV: 17-43), mg/dL	24	20 (17-26)	21	20 (15-24)	.45
Sodium (RV: 135-145), mEq/L	32	137 (136-139)	28	138 (136-140)	.20
Potassium (RV: 3.5-5), mEq/L	32	4.7 (4.5-4.9)	28	4.7 (4.3-5)	.78
Urine osmolality (RV: >600), mOsm/kg	11	736 (288-1026)	10	673 (410-925)	.71
Plasma osmolality (RV: 275-300), mOsm/kg	11	301 (297-305)	9	298 (296-302)	.33

Abbreviations: IQR, interquartile range; RV, reference values.

SI conversion factors: To convert corticotropin to picomoles per liter, multiply by 0.22; cortisol to nanomoles per liter, multiply by 27.588; free thyroxine to picomoles per liter, multiply by 12.87; IGF-1 to nanomoles per liter, multiply by 0.13; IGFBP-3 to μg/dL, multiply by 0.13; glucose to millimoles per liter, multiply by 0.0555; insulin to picomoles per liter, multiply by 6.945; plasma and urine osmolality to millimoles per kilogram, multiply by 1.0; potassium to millimoles per liter, multiply by 1.0; prolactin to micrograms per liter, multiply by 1; sodium to millimoles per liter, multiply by 1.0; urea to millimoles per liter, multiply by 0.357.

^a^ Data were compared using the Mann-Whitney U test. The sample size variation in some features reflects the availability of specific information.

Neonates with severe microcephaly presented with significantly shorter lengths at birth by median (IQR) *z* score than those with nonsevere microcephaly (−1.9 [−2.5 to −1.0] vs −0.3 [−1.0 to 0]; *P* < .001) (Table 1). This difference did not persist at 27 months of age (−1.6 [−2.3 to −0.3] vs −2.9 [−4.0 to −1.2]; *P* = .06). In all, 22 of 50 (44.0%) patients had a length *z* score more than 2 SDs below the mean at 27 months. However, when we compared the number of patients who remained short and had a length *z* score more than 2 SDs below the mean at 27 months of age, there was no statistically significant difference between the patients with severe vs nonsevere microcephaly (14 of 25 patients [56.0%] vs 8 of 25 patients [32.0%]; *P* = .09) patients. Median (IQR) circulating levels of IGF-1 were higher in patients with severe microcephaly (162 [118-226] ng/mL vs 129 [91-163] ng/mL [to convert to nanomoles per liter, multiply by 0.131]; P = .01), but levels of IGFBP3 were not statistically significantly different. The levels of IGF-1 and IGFBP3, however, were within the reference range for all but 1 patient (Table 2; eTable 2 in the Supplement); this patient showed low IGF-1 concentrations (43.08 ng/mL) but had IGFBP3 levels in the reference range. A growth hormone stimulation test was not performed for this patient.

A simultaneous evaluation of plasma and urine osmolality was performed for 21 patients. We did not perform these evaluations for all patients because of the difficulty in collecting urine samples from many patients. Clinically, the mothers of all patients denied polyuria or the need for frequent diaper replacement. No patients had a history of dehydration, and all their sodium levels were in the reference range (Table 2; eTable 2in the Supplement). Of 21 patients evaluated, 13 patients (61.9%) had a urine osmolality higher than 600 mOsm/kg, excluding a diagnosis of diabetes insipidus. Low urine osmolality (<300 mOsm/kg) and high plasma osmolality (>300 mOsm/kg [to convert to millimoles per kilogram, multiply by 1.0]) were found in 1 patient. The patient with confirmed diabetes insipidus had levels of glucose, urea, and sodium in the reference ranges. Urine osmolality less than 300 mOsm/kg and plasma osmolality less than 300 mOsm/kg were found in 3 patients (14.3%). A water deprivation test was indicated for these patients, but their caregivers did not consent to having new samples taken. Plasma osmolality was within the upper reference range for most patients, being greater than 300 mOsm/kg for 10 out of 20 patients (50.0%). However, in 6 of these patients, urine osmolality was higher than 600 mOsm/kg, which excluded diabetes insipidus. Thus, in this subset of patients, the evaluation of basal urine and plasma osmolality excluded diabetes insipidus for 13 patients, confirmed it for 1 patient (4.8%), and suggested it for 3 patients. For the remaining patients, the basal laboratory evaluation alone did not allow for any conclusions.

Using CNS CT scans, we found severe defects in most patients (61 patients [93.8%]), including calcifications in 61 patients, lissencephaly in 38 patients (58.5%), cerebellar atrophy or hypoplasia in 16 patients (24.6%), cerebral atrophy in 41 patients (63.1%), hydrocephalus in 9 patients (13.8%), ventriculomegaly in 42 patients (64.6%), and agenesis or hypoplasia of the corpus callosum in 25 patients (38.5%) (Figure and Table 3). We found no hormone level differences when comparing patients with vs without corpus callosum defects, except for higher median (IQR) IGF-1 levels among those with defects (195 [109-220] ng/mL vs 141 [101-164] ng/mL; *P* = .02) (Table 4). Optic nerve atrophy was observed in 38 patients (58.5%). We also compared pituitary function in patients according to the presence or absence of optic nerve atrophy, and no differences were observed (eTable 3 in the Supplement).

**Figure.**
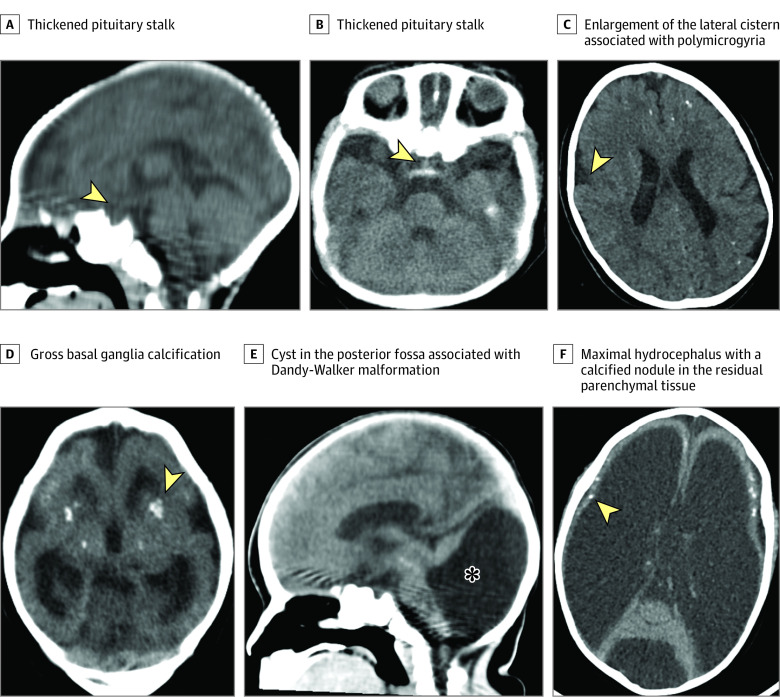
Noncontrast Axial Computed Tomographic (CT) Images and Sagittal Reconstructions of CT Images Panels B, C, D, and F are noncontrast axial CT images. Panels A and E are sagittal reconstructions of CT images. Arrows and asterisk indicate specified features.

**Table 3.** Frequency of Central Nervous System Defects Discovered by Computed Tomography

Condition^a^	Total (N = 65)		With severe microcephaly (n = 36)		With mild or moderate microcephaly (n = 29)		*P* value
	Sample size, No.	No. (%)	Sample size, No.	No. (%)	Sample size, No.	No. (%)	
Calcification	65	61 (93.8)	36	35 (97.2)	29	26 (89.7)	.31
Localization of calcification							
Subcortical	48	21 (43.8)	23	14 (60.9)	25	7 (28.0)	.05
Periventricular	48	20 (41.7)	23	6 (26.1)	25	14 (56.0)	.07
Basal nuclei	48	14 (29.2)	23	5 (21.7)	25	9 (36.0)	.44
All	48	14 (29.8)	23	9 (39.1)	25	5 (20.0)	.29
Ventriculomegaly	65	42 (64.6)	36	28 (77.8)	29	14 (48.3)	.02
Hydrocephaly or hydranencephaly	65	9 (13.8)	36	8 (22.2)	29	1 (3.4)	.03
Cerebral atrophy	65	41 (63.1)	36	26 (72.2)	29	15 (51.7)	.14
Lissencephaly or pachygyria	65	38 (58.5)	36	25 (69.4)	29	13 (44.8)	.08
Cerebellar hypoplasia	65	16 (24.6)	36	13 (36.1)	29	3 (10.3)	.02
Agenesis of the corpus callosum or hypoplasia	65	25 (38.5)	36	16 (44.4)	29	9 (31.0)	.39

^a^ Data were compared using the Fisher exact test.

**Table 4.** Hormonal and Biochemical Profiles of Patients by Presence or Absence of Corpus Callosum Defects

Characteristic^a^	Median (IQR)		*P* value
	Without corpus callosum defect (n = 40)	With corpus callosum defect (n = 25)	
Age, mo	26 (25-28)	27 (25-29)	.37
Free thyroxine (RV: 0.54-1.43), ng/dL	0.9 (0.9-1.1)	0.9 (0.8-1.0)	.88
Thyrotropin (RV: 0.5-6.6), mIU/L	2.6 (2.0-3.5)	2.3 (1.6-3.9)	.48
Cortisol (RV: 5-18), μg/dL	8.1 (6.7-9.9)	7.4 (7.1-9.4)	.92
Corticotropin (RV: 7- 3), pg/mL	13 (8-20)	16 (11-19)	.71
IGF-1 (RV: 49-270), ng/mL	141 (101-164)	195 (109-220)	.02
IGFBP-3 (RV: 0.8-3.9), μg/mL	3.1 (2.3-3.7)	3.3 (2.6-4.1)	.24
Prolactin (RV: 2.6-25), ng/mL	9 (6-12)	7 (4-11)	.61
Glucose (RV: 70-100), mg/dL	81 (77-90)	80 (70-86)	.22
Insulin (RV: 2.5-25), mIU/L	4.8 (3.2-11)	7.5 (4.5-18)	.15
Urea (RV: 17-43), mg/dL	19 (15-23)	21 (17-26)	.41
Sodium (RV: 135-145), mEq/L	138 (136-139)	138 (136-139)	.75
Potassium (RV: 3.5-5), mEq/L	4.7 (4.4-4.9)	4.7 (4.4-5)	.72
Plasma osmolality (RV: >600), mOsm/kg	299 (297-304)	301 (299-304)	.43
Urine osmolality (RV: 275-300), mOsm/kg	646 (410-1022)	736 (288-964)	.82

Abbreviations: IQR, interquartile range; RV, reference values.

SI conversion factors: To convert corticotropin to picomoles per liter, multiply by 0.22; cortisol to nanomoles per liter, multiply by 27.588; free thyroxine to picomoles per liter, multiply by 12.87; IGF-1 to nanomoles per liter, multiply by 0.13; IGFBP-3 to μg/dL, multiply by 0.13; glucose to millimoles per liter, multiply by 0.0555; insulin to picomoles per liter, multiply by 6.945; plasma and urine osmolality to millimoles per kilogram, multiply by 1.0; potassium to millimoles per liter, multiply by 1.0; prolactin to micrograms per liter, multiply by 1; sodium to millimoles per liter, multiply by 1.0; urea to millimoles per liter, multiply by 0.357.

^a^ Data were compared using the Mann-Whitney *U* test.

Some features were not assessed for the entire cohort; eTable 4, eTable 5, and eTable 6 in the Supplement show the missing values. None of the study participants were lost to follow-up.

## Discussion

Severe microcephaly and other brain defects are common sequelae of some congenital infections and are particularly frequent with CZS.^4,16^ Hypopituitarism may occur in patients with severe CNS lesions, particularly midline brain defects.^17,18,19^ In this cohort study, we evaluated a large group of children with microcephaly caused by CZS in the northeast region of Brazil, which was the epicenter of this epidemic from 2015 to 2016.

Most patients in our study presented with severe impairment of mobility. Congenital contractures were observed in 28.1% of patients, and 96.9% of patients presented with gross motor developmental delay. Seizures were also frequent. Among all patients, 12.3% presented with seizures in the neonatal period and 72.3% presented with seizures at 27 months. Our central hypothesis was that patients with congenital Zika infection, particularly those with severe microcephaly, with or without midline brain defects, would present with hypopituitarism. Most patients did not have past or present clinical signs of hypopituitarism, and none had biochemical features of thyrotropin or growth hormone deficiency. However, 9 patients (13.8%) presented with cortisol levels less 5 μg/dL, suggesting adrenal insufficiency. Diabetes insipidus was excluded for most but not all patients. We did not observe any associations between impaired pituitary function and optic nerve atrophy or agenesis or hypoplasia of the corpus callosum, present in 58.5% and 38.5% of our patients, respectively. Of note, the association between midline brain defects, such as septo-optic dysplasia and hypopituitarism, is well documented.^20,21,22^

In 2021, Gonçalves et al^23^ evaluated 30 patients with CZS who underwent endocrine evaluations. In general, the results showed frequent short stature but absence of hypopituitarism; these results overlap with ours. However, as those patients' ages were higher than ours, Gonçalves et al^23^ found signs of early adrenarche in 27% of the patients. Notably, early adrenarche has frequently been described in patients born small for their gestational age. Similar to our results, Gonçalves et al found a high prevalence of short stature. Moreover, they described the occurrence of primary hypothyroidism in 2 patients.^23^ In general, only case reports or small studies of patients with congenital infections investigating hypothalamic-pituitary function in children have been published to date.^24,25,26^ None of these studies have investigated patients with CZS. In these types of studies, the temporal causal relationship for hormone deficiencies is difficult to establish when present. In a case report by Chan et al,^24^ an autopsy of these patients showed holoprosencephaly and cerebellar hypoplasia in addition to pituitary, adrenal, and thyroid hypoplasia.

A study^27^ of patients with severe congenital toxoplasmosis found endocrine diseases in 5 out of 17 patients (29.4%). Of these patients, 2 had pan-hypopituitarism, 1 had growth deficiency and hypogonadism, 1 had growth deficiency and developed precocious puberty, and 1 had hypothyroidism and type 1 diabetes. Third-ventricle hydrocephaly and optic nerve atrophy were observed in all patients. The median age at diagnosis was 12 years. These findings highlight the importance of prospective evaluations of patients investigated in the present study, given that endocrine diseases may develop later in childhood.^27^

In our study, 44.0% of patients had a length *z* score more than 2 SDs below the mean at age 27 months. There was a trend toward a shorter length in the group with severe microcephaly. However, most of these patients were born short and did not show catch-up growth in the first 2 years of life. Growth hormone deficiency is defined by clinical signs, ectopic neurohypophysis, hypoplastic adenohypophysis, septo-optic dysplasia, and the presence in neonates and very young children of at least two additional pituitary hormone deficiencies. Clinical signs include neonatal hypoglycemia, jaundice, and micropenis in boys.^28^ The absence of typical signs associated with multiple pituitary hormone deficiencies in our patients makes the diagnosis of growth hormone deficiency unlikely.

The reference range levels of IGF-1 and IGFBP3 in all but 1 patient at the age of 27 months in our study reinforce this conclusion. However, it should be noted that the levels of IGF-1 and IGFBP3 are physiologically low in the first 2 years of life, and it is frequently difficult to differentiate between patients who have reference range levels and those who have deficiencies. We did not perform a growth hormone stimulation test, considered the mainstay of diagnostic investigations for growth hormone deficiency in older patients,^29^ for the patients with low IGF-1 and IGFBP3 levels. Thus, our results suggest that growth hormone deficiency was not common in patients with CZS and microcephaly. These patients presented with prenatal growth impairment and a lack of postnatal catch-up, which is likely not associated growth hormone deficiency. Interestingly, at the age of 27 months, patients in the group with severe microcephaly presented higher IGF-1 levels, although the levels were still within the reference range. This finding could indicate the existence of some degree of IGF-1 resistance. Indeed, patients in this group were shorter at birth; however, this difference did not persist at 27 months.

Severe neurodevelopmental delay is common in patients with CZS and microcephaly,^4,30^ and the occurrence of hypothyroidism, an easily treatable condition, could worsen this scenario. However, we found that this condition did not occur often in these patients, as the patients’ FT_4_ and thyrotropin levels were in the reference range. Transient or definitive central hypothyroidism has been reported in neonates with encephalitis caused by human parechovirus type 3.^31^

For patients with congenital hypopituitarism, adrenal insufficiency is the most life-threatening condition.^32^ The clinical signs of central adrenal insufficiency vary considerably depending on the patient’s age and the amount and severity of related pituitary deficiencies.^33^ None of the 9 patients in our study with low cortisol levels presented with clinical signs of adrenal insufficiency. However, most of the patients (96.9%) had significant motor neurodevelopmental delays, which may impair the clinical diagnosis. Interestingly, 8 of these patients presented with a history of seizures at the time of their endocrine evaluations and were receiving anticonvulsant treatment. The occurrence of seizures has been attributed to brain malformations. Some anticonvulsants, such as oxcarbazepine, accelerate cortisol elimination via cytochrome P450 induction and decrease plasma total cortisol levels.^34^ No patients in this study presented with hypoglycemia or low cortisol levels simultaneously, however. Moreover, no hypoglycemia was documented during the seizure episodes. Therefore, these seizures were attributed to severe CNS malformations associated with congenital Zika virus infection syndrome.

A central adrenal insufficiency diagnosis relies on the demonstration of low morning basal cortisol concentrations and insufficient cortisol release during a dynamic test with a stimulating agent.^33,35,36^ An extremely low morning basal cortisol level (ie, <3 μg/dL) indicates adrenal insufficiency.^33,36^ Using 3 μg/dL as a cutoff value, 1 patient in this study would have a diagnosis of central adrenal insufficiency confirmed by basal cortisol measurement. However, using the cut-off value of 3.9 μg/dL established by Maguire et al (2008),^35^ which has a sensitivity of 83% and a specificity of 99%, 4 patients (6.2%) would have a diagnosis of central adrenal insufficiency. In most of the patients (75.4%) in this study, the basal cortisol levels ranged from 5 to 13 μg/dL. Although not mandatory, only a cortisol stimulatory test would exclude partial central adrenal insufficiency in these patients.^33,35,36^

We simultaneously evaluated plasma and urine osmolality in a subset of 21 patients. None of these patients presented with a history of dehydration or clinically relevant polyuria. Moreover, their sodium levels were within the reference range. We experienced difficulties collecting blood and urine samples from the remaining patients to measure osmolality. Typically, these patients are very spastic, their water ingestion rate is generally low, and their diet is usually very thick to prevent bronchoaspiration. To collect these blood and urine samples, these patients were evaluated for at least 6 hours, and most were found to have low urinary volumes. We excluded diabetes insipidus in 61.9% of the patients with a urine osmolality higher than 600 mOsm/kg.^37,38^ One patient with a urine osmolality lower than 300 mOsm/kg, a plasma osmolality higher than 300 mOsm/kg, and reference range urea and glucose levels was confirmed to have diabetes insipidus. Moreover, 3 patients (14.3%) presented high plasma osmolality values but urine osmolality values between 300 and 600 mOsm/kg, which can occur in patients with partial diabetes insipidus.^37,38^ A water deprivation test was indicated for these patients, but the test was not approved by their parents. A 2015 study^39^ with a small series of patients with CNS infections did not find any instances of diabetes insipidus. However, diabetes insipidus is common in patients with severe midline defects, including holoprosencephaly.^40^

Our study adds new relevant clinical information on children with microcephaly and a range of CNS malformations associated with congenital Zika virus infection. To our knowledge, this is one of the first studies to assess the hypothalamic-pituitary function in these patients, and we found that hypopituitarism occurred in some patients. Moreover, the Zika virus epidemic occurred recently, so the long-term outcomes associated with CZS infections are not yet known. Thus, biochemical investigations and a prospective clinical evaluation of the growth and pubertal development among these patients are warranted. The evolving literature in this field continues to show additional long-term consequences as these patients develop.^30,41,42,43^

### Limitations

This study has several limitations. The epidemiological data were obtained retrospectively, the Zika virus infections were not confirmed with immunoassays, and the patients were followed up in referral centers, which may have biased the selection process toward patients with more severe illness. Although the same physician performed all clinical examinations, significant spasticity among some patients may have affected the precision of length measurements. Another important limitation is that we did not perform stimulatory tests to detect growth hormone or corticotropin deficiencies. However, with regard to growth hormone deficiencies, all patients had IGF-1 and IGFBP3 levels in the reference range, which made any growth hormone deficiency diagnoses unlikely. An additional limitation is the absence of magnetic resonance imaging (MRI) scans for these patients, as MRI is the gold standard for evaluating the pituitary gland, hypothalamus, and surrounding structures.

## Conclusions

This study found that there were no growth hormone or thyrotropin deficiencies in children with CZS-associated microcephaly. However, central adrenal insufficiency and diabetes insipidus were detected in a few patients. Nevertheless, it should be noted that hormonal defects may appear many years after the infection occurs, similar to what has been reported in patients with other types of CNS infections. Therefore, prospective clinical and biochemical endocrine follow-up studies of patients with CZS are recommended.

## Notes

Supplement. **eTable 1.** Mothers’ Characteristics by Degree of Microcephaly of Their Children **eTable 2.** Detailed and Individualized Clinical, Hormonal, and Biochemical Patient Profiles by Presence or Absence of Optic Nerve Atrophy or Hypoplasia **eTable 3.** Hormonal and Biochemical Patient Profiles by Presence or Absence of Optic Nerve Atrophy or Hypoplasia **eTable 4.** Missing Values in Clinical and Developmental Characteristics **eTable 5.** Missing Values in Hormonal Profile **eTable 6.** Missing Values in Computed Tomography Scan Results
