# Supplementary material for: Clinical and Biochemical Features of Hypopituitarism Among Brazilian Children With Zika Virus–Induced Microcephaly
Source: JAMA Netw Open. 2021 May 13;4(5):e219878. doi: 10.1001/jamanetworkopen.2021.9878 (PMC8120328; doi:10.1001/jamanetworkopen.2021.9878)
Supplement: Supplement. — eTable 1. Mothers’ Characteristics by Degree of Microcephaly of Their Children eTable 2. Detailed and Individualized Clinical, Hormonal, and Biochemical Patient Profiles by Presence or Absence of Optic Nerve Atrophy or Hypoplasia eTable 3. Hormonal and Biochemical Patient Profiles by Presence or Absence of Optic Nerve Atrophy or Hypoplasia eTable 4. Missing Values in Clinical and Developmental Characteristics eTable 5. Missing Values in Hormonal Profile eTable 6. Missing Values in Computed Tomography Scan Results [file jamanetwopen-e219878-s001.pdf]

Supplemental Online Content

Ferreira LL, Aguilar Ticona JP, Silveira-Mattos PS, et al. Clinical and biochemical features of hypopituitarism among Brazilian children with Zika virus–induced microcephaly. *JAMA Netw Open*. 2021;4(5):e219878. doi:10.1001/jamanetworkopen.2021.9878

eTable 1. Mothers' Characteristics by Degree of Microcephaly of Their Children
eTable 2. Detailed and Individualized Clinical, Hormonal, and Biochemical Patient Profiles by Presence or Absence of Optic Nerve Atrophy or Hypoplasia
eTable 3. Hormonal and Biochemical Patient Profiles by Presence or Absence of Optic Nerve Atrophy or Hypoplasia
eTable 4. Missing Values in Clinical and Developmental Characteristics
eTable 5. Missing Values in Hormonal Profile
eTable 6. Missing Values in Computed Tomography Scan Results

This supplemental material has been provided by the authors to give readers additional information about their work.

© 2021 Ferreira LL et al. *JAMA Network Open.*

eTable 1. Mothers' Characteristics by Degree of Microcephaly of Their Children

	n	Total	n	Mild or Moderate Microcephaly	n	Severe Microcephaly	p-value
Age, median (IQR), years	63	27.0 [21.0, 32.0]	29	28.0 [22.0, 32.0]	34	25.5 [20.2, 30.0]	.09
In a stable relationship (%) [a]	64	37 (57.8)	29	16 (55.2)	35	21 (60.0)	.89
Schooling (%)							.36
Incomplete elementary	63	7 (11.1)	29	5 (17.2)	34	2 (5.9)	
Elementary	63	3 (4.8)	29	1 (3.4)	34	2 (5.9)	
Incomplete high school	63	17 (27.0)	29	5 (17.2)	34	12 (35.3)	
High school	63	31 (49.2)	29	15 (51.7)	34	16 (47.1)	
Graduate	63	5 (7.9)	29	3 (10.3)	34	2 (5.9)	
From a rural area (%)	64	22 (34.4)	29	8 (27.6)	35	14 (40.0)	.44
Prenatal care, median (IQR)	64	8.0 [6.8, 9.0]	29	8.0 [7.0, 9.0]	35	8.0 [6.0, 9.0]	.65
Smoking during the pregnancy (%)	64	4 (6.2)	29	1 (3.4)	35	3 (8.6)	.75
Alcohol use during the pregnancy (%)	64	7 (10.9)	29	1 (3.4)	35	6 (17.1)	.18
Drugs use during the pregnancy (%)	64	1 (1.6)	29	0 (0.0)	35	1 (2.9)	1.00
Nutritional suppl., folic acid (%)	63	3 (4.8)	29	2 (6.9)	34	1 (2.9)	.89
Consanguineous parents (%)	64	1 (1.6)	29	0 (0.0)	35	1 (2.9)	1.00
Previous children with MC (%)	64	3 (4.7)	29	1 (3.4)	35	2 (5.7)	1.00
Symptoms during pregnancy (%)							
Fever	64	34 (53.1)	29	15 (51.7)	35	19 (54.3)	1.00
Rash	64	49 (76.6)	29	23 (79.3)	35	26 (74.3)	.86
Arthralgia	63	28 (44.4)	29	13 (44.8)	34	15 (44.1)	1.00
Pruritus	63	39 (61.9)	29	20 (69.0)	34	19 (55.9)	.42
Conjunctival injection	63	15 (23.8)	29	8 (27.6)	34	7 (20.6)	.72
Myalgia	63	23 (36.5)	29	12 (41.4)	34	11 (32.4)	.63
Month of symptoms (median [IQR])	54	3.0 [2.0, 4.0]	24	3.0 [3.0, 4.2]	30	3.0 [2.0, 3.0]	**.02**

© 2021 Ferreira LL et al. *JAMA Network Open.*

Trimester of symptoms							**.02**
First trimester	54		24	13 (54.2)	30	26(86.7)	
Second and third trimester	54		24	11 (45.8)	30	4(13.3)	
Self-report of Zika-like symptoms in sexual partner (%)	63	9 (14.3)	29	1 (3.4)	34	8 (23.5)	.06
Caesarean delivery (%)	64	36 (56.2)	29	16 (55.2)	35	20 (57.1)	1.00

[a]Being in a stable relationship (marital status option) was defined as cohabiting or being married.

© 2021 Ferreira LL et al. *JAMA Network Open.*

eTable 2. Detailed and Individualized Clinical, Hormonal, and Biochemical Patient Profiles by Presence or Absence of Optic Nerve Atrophy or Hypoplasia

N	sex	MCS/NS	GA mo	Neo seiz	Lenght SD	HC SD	Age mo	FT4 ng/dL	TSH mUI/mL	Cort mcg/dL	Insulin mUi/mL	ACTH pg/mL	IGF-1 ng/mL	IGFBP-3 mcg/dL	Prolactin ng/mL	Glucose mg/dL	Urea mg/dL	Na mEq/L	K mEq/L	Posm mOsm/kg	Uosm mOsm/kg	Seizures 1y
1	F	S	39	no	-1,87	-6,54	23	0,89	1,55	8,25	13,64	18,5	145,7	2,36	8,42	65	26	136	5,5	293	1068	yes
2	M	S	39	no	0,86	-3,00	26	0,78	1,60	2,2	5,54	25,38	312,4	3,6	15,56	80	20	136	5,3	285	736	yes
3	M	S	40	no	-1,12	-7,48	24	0,86	1,90	7,7	7,46	13,47	107,6	2,65	4,24	90	29	136	5,1	304	975	yes
4	F	S	37	no	0,06	1,28	23	0,84	1,95	5,1	12,61	12,52	211,9	4,1	9,06	100	14	136	4,5	301	288	yes
5	M	S	34	no	-4,10	-9,67	22	1,05	2,72	4,6	4,08	6,7	162,8	2,62	7,59	90	18	137	5,3	304	413	yes
6	M	S	38	no	-1,15	-4,21	25	0,92	1,91	9,1	11,72	19,66	285,3	4,44	7,17	86	39	138	4,8	305	1166	no
7	F	NS	39	yes	-1,66	-6,17	23	0,86	2,37	8,7	20,54	26,49	128,7	2,92	7,78	70	18	138	4,3	306	925	yes
8	M	NS	34	no	-1,52	-4,92	25	0,86	2,64	3,3	1,98	12,88	146	1,52	9,07	72	10	137	4,4	298	166	yes
9	M	NS	41	no	0,73	-4,84	25	1,17	3,02	12,1	8,28	9,6	174,6	3,48	11,67	81	15	136	4,5	NA	410	no
10	F	S	41	no	-3,19	-8,75	27	0,74	5,28	7,1	2,71	17,42	109,2	2,67	6,32	85	19	135	4,4	299	964	yes
11	F	S	40	no	-3,05	-9,85	26	0,95	3,92	8,74	6,09	22,45	119,9	0,717	7,87	73	21	136	4,6	345	1164	yes
12	M	NS	37	no	5,62	-6,95	23	1,02	3,07	10,6	1,98	23,33	214,3	3,33	14,28	76	24	141	5,5	296	630	yes
13	F	S	41	no	-3,93	-8,46	24	1	1,97	13,0	11,14	9,32	299,9	4,33	5,63	67,3	NA	136	4,99	301	1026	yes
14	M	S	40	no	-1,92	-7,40	25	1,05	3,44	6,7	16,91	4,2	147	4,21	12,19	82,3	NA	138	4,3	293	646	yes
15	F	NS	38	no	-1,32	-3,38	26	1,06	1,98	14,7	5,48	33,27	162,9	3,12	18,88	92,9	NA	139	4,3	302	533	yes
16	M	NS	NA	no	-3,15	-6,15	25	1	2,64	7,56	4,76	7,74	47,22	1,62	12,16	88,9	NA	141	4,3	301	369	yes
17	M	NS	40	no	NA	NA	24	1,32	2,63	12,0	7,99	19,6	73,49	3,16	8,25	80,4	NA	139	4,8	311	716	no
18	M	S	41	no	NA	NA	25	0,79	3,35	8,47	22,03	7,24	246,2	4,12	25,33	72,9	NA	139	4,7	296	169	yes
19	M	NS	38	no	-1,99	-5,96	27	0,84	2,26	6,7	15,03	8,11	216,6	3,63	15,51	110	15	138	4,3	296	1022	no

© 2021 Ferreira LL et al. *JAMA Network Open.*

20	F	NS	41	no	0,11	-0,17	26	0,87	2,12	8,3	1,39	11	133,7	3,78	7,1	81	15	133	5,3	295	788	no
21	M	S	39	yes	-1,34	-7,47	29	0,84	0,68	8,8	6,22	16,9	220,3	4,69	3,7	NA	NA	NA	NA	297	162	yes
22	M	NS	37	no	-1,61	-5,11	24	0,8	5,02	8,4	1,65	13,18	147,1	3,04	11,43	77	19	137	5,4	NA	NA	no
23	M	S	37	no	0,32	2,07	25	1,24	4,53	9,4	8,73	13,76	195,9	3,47	3,72	79	8	140	4,7	NA	NA	no
24	F	S	37	yes	-4,39	-8,35	26	1,03	4,11	6,9	5,84	57,1	288,9	4,63	19,46	90	26	139	4,5	NA	NA	yes
25	M	NS	40	no	-4,46	-4,04	26	0,66	3,18	8,6	2,54	15,22	99,6	1,78	3,35	74	17	136	4,7	NA	NA	no
26	F	NS	40	no	-0,26	-3,86	27	1,08	3,46	4,4	3,85	35,76	136,2	3,21	7,07	90	22	138	5,3	NA	NA	yes
27	F	NS	38	no	-0,83	-5,99	27	0,94	3,37	8,02	1,06	17,72	58,18	3,8	5,75	85	14	138	4,8	NA	NA	yes
28	M	S	34	no	-4,79	-4,79	25	1,01	0,96	7,15	22,24	17,99	296,3	4,33	14,51	85	27	138	4,8	NA	NA	yes
29	F	S	38	no	-4,26	-7,72	27	0,75	1,01	11,0	2,21	12,99	128,9	2,74	3,44	78	19	137	4,4	NA	NA	yes
30	M	S	39	no	-1,76	-6,34	30	0,71	1,84	6,6	20,94	4,7	69,83	1,47	5,38	96	16	140	4,9	NA	NA	yes
31	F	S	38	no	-5,20	-5,76	12	0,86	3,00	7,1	10,82	18,54	160,8	4,6	12,22	90	15	138	4,9	NA	NA	yes
32	M	NS	37	no	-4,72	-7,21	28	0,69	2,32	6,7	12,94	11,46	190,4	3,1	10,62	98	26	141	4,1	NA	NA	yes
33	F	NS	32	yes	-2,38	-4,91	27	0,82	4,56	4,9	1,95	11,19	88,04	1,86	4,54	76	25	139	5,3	NA	NA	yes
34	F	S	38	no	NA	NA	27	0,93	1,12	7,22	6,89	12,87	115,7	2,2	4	62	26	138	4,9	NA	NA	yes
35	M	S	39	no	-3,96	-8,09	29	1,01	1,34	7,4	18,03	8,36	230,8	4,26	11,78	62	26	135	4,6	NA	NA	yes
36	F	NS	NA	no	0,22	-4,88	28	0,96	4,72	11,3	1,65	11,95	101,7	3,42	15,46	86	23	137	4,4	NA	NA	yes
37	F	NS	39	no	-0,12	-3,27	27	0,93	0,89	3,5	4,48	2,7	204,9	3,74	7,37	88	27	138	4,7	NA	NA	yes
38	M	NS	40	no	-5,16	-3,13	29	0,86	4,97	11,3	10,55	18,09	96,11	2,56	8,58	76	27	141	4,2	NA	NA	no
39	F	S	38	no	-2,36	NA	29	1,22	3,55	4,4	24,02	12,55	169	2,26	9,45	87,3	NA	137	4,7	NA	NA	yes
40	M	S	36	no	-1,20	-5,12	28	1,19	1,74	5,5	9,62	13,01	194,1	3,3	8,96	81	20	141	4,3	NA	NA	yes
41	M	S	38	yes	NA	NA	27	0,93	2,85	7,76	4,75	19,14	81,26	1,84	7,4	68	33	145	4,6	NA	NA	yes

© 2021 Ferreira LL et al. *JAMA Network Open.*

42	M	NS	39	no	-2,67	-5,71	27	1,43	0,87	8,31	3,01	15,14	112,6	3,46	11,67	79	14	138	5,1	NA	NA	yes
43	F	NS	40	no	1,63	-2,62	25	0,74	3,98	8,49	16,76	12,45	151,8	1,32	8,93	93	24	136	4,7	NA	NA	yes
44	F	S	39	no	-3,77	-4,55	23	0,98	1,35	4	8,01	29,8	192,7	4,15	7,44	86	24	136	4,4	NA	NA	yes
45	M	S	41	no	NA	NA	24	0,6	5,42	3,2	4,41	9,31	168,4	2,66	23,8	79	23	135	5,3	NA	NA	yes
46	F	S	40	yes	NA	NA	29	0,99	3,08	8,09	28,75	16,42	159,6	2,62	11,34	70	14	140	4,4	NA	NA	yes
47	M	NS	39	no	NA	NA	31	1,01	4,19	10,0	8,34	16,45	194,9	3,55	3,6	81,5	NA	136	4,5	NA	NA	yes
48	F	NS	36	no	-2,32	-3,62	31	0,92	3,89	7,31	1,35	17,34	90,94	2,26	3,81	73	21	141	4,1	NA	NA	yes
49	M	S	40	no	-1,74	-10,95	30	1,09	2,18	6,85	4,76	8,08	113,1	2,23	11,1	81	14	140	4,4	NA	NA	yes
50	M	S	38	yes	-0,12	NA	31	0,88	1,04	16,2	31,49	26,87	271,2	3,83	9,54	82	18	139	3,9	NA	NA	yes
51	F	S	39	no	NA	NA	30	0,9	1,57	6,78	4,75	17,46	150,4	3,34	3,6	NA	27	135	4,9	NA	NA	no
52	M	S	36	yes	-4,73	-3,87	30	1,2	2,02	9,97	4,42	46,14	145,2	3,67	19,4	85	19	137	4,6	NA	NA	yes
53	F	NS	NA	no	-1,17	-2,05	32	1,09	2,39	5,4	0,59	18,81	78,34	0,619	16,13	69,9	NA	141	4,8	NA	NA	no
54	M	NS	41	no	-1,15	-3,05	30	0,92	2,46	23,4	11,82	20,4	109	4,3	33,6	111	20	139	4,6	NA	NA	no
55	M	NS	NA	no	-2,16	-5,22	28	0,83	2,12	14,5	1,94	30,24	43,01	1,36	6,88	60	13	130	4,9	NA	NA	yes
56	F	S	41	no	NA	NA	24	0,93	3,71	15,2	3,55	26,62	61,75	1,98	12,65	86,7	NA	136	4,9	NA	NA	yes
57	M	S	34	no	-3,56	-6,85	31	0,86	2,78	16,9	20,81	21,8	68,1	2,79	3	NA	NA	NA	NA	NA	NA	yes
58	M	S	37	no	NA	NA	32	1,2	4,69	10,9	6,75	19,51	291,7	5,15	4,47	81,9	NA	136	5,1	NA	NA	no
59	M	S	36	no	-2,85	-7,00	30	1,21	2,33	3,9	12,98	7,23	122,1	2,53	6,78	92,9	NA	139	4,5	NA	NA	no
60	M	NS	NA	no	NA	NA	28	1,06	1,02	6,9	4,04	5,5	184,2	3,41	5,26	68,7	NA	137	3,9	NA	NA	no
61	F	NS	39	no	-1,81	-4,63	26	0,72	1,94	9,83	4,79	8,1	84,84	1,59	3,3	78,6	NA	146	4,7	NA	NA	no
62	M	NS	40	no	NA	NA	NA	1,07	3,95	7,3	2,41	8,3	103	3,19	5,5	NA	NA	NA	NA	NA	NA	yes

© 2021 Ferreira LL et al. *JAMA Network Open.*

63	F	S	40	no	NA	NA	NA	0,83	2,68	7,5	3,19	8,1	108,1	2,74	5,7	NA	NA	NA	NA	NA	NA	yes
64	M	S	38	no	NA	NA	NA	0,98	4,55	5,2	3,08	10,1	186,8	NA	5,7	71	NA	138	4,7	NA	NA	no
65	F	S	38	no	NA	NA	NA	0,81	3,10	13,4	2,07	5,1	160	3,41	3,7	NA	NA	NA	NA	NA	NA	yes

M= male; F= female; NA= not available; MC= microcephaly; S= severe; NS= non severe; GA= gestational age; mo= months; HC= head circumference; SD= standard deviation. For SI units, multiply by 12.87 (FT4), 27.58 (cortisol), 0.22 (ACTH), 0.13 (IGF-1), 1 (IGFBP-3), 1 (prolactin), 0.05 (glucose), 6.94 (insulin), 0.35 (urea), 1 (Na), 1 (K), and 1 (plasma and urine osmolality).

© 2021 Ferreira LL et al. *JAMA Network Open.*

eTable 3. Hormonal and Biochemical Patient Profiles by Presence or Absence of Optic Nerve Atrophy or Hypoplasia

	**Normal Optic Nerve (n= 27) Median (IQR)**	**Optic Nerve Atrophy (n= 38) Median (IQR)**	**P value**
Age (months)	26.5 (25 – 29)	27 (25 – 29)	0.85
Free Thyroxine (ng/dL; RV: 0.54 – 1.43)	1.01 (0.8 – 1)	0.9 (0.8 – 1)	0.18
TSH (mUI/L; 0.5 – 6.6)	2.4 (1.9 – 3.1)	2.6 (1.8 – 3.9)	0.74
Cortisol (mcg/dL; VR: 5 - 18)	7.3 (6.1 – 8.9)	7.7 (6.7 – 9.9)	0.57
ACTH (pg/mL; 7- 3)	13 (8.3 – 20)	14 (9.5 – 19)	0.75
IGF-1 (ng/mL; RV: 49 - 270)	168 (132 – 196)	137 (97 – 191)	0.07
IGFBP-3 (mcg/mL; RV: 0.8 – 3.9)	3.3 (2.9 – 3.6)	2.6 (2.2 – 3.8)	0.19
Prolactin (ng/mL; RV: 2.6 - 25)	11 (7 – 15)	7 (4 – 12)	0.05
Glucose (mg/dL; RV: 70 - 100)	81 (74 – 90)	79 (72 – 86)	0.32
Insulin (mIU/L; RV: 2.5 - 25)	5.5 (2.9 – 12.3)	5.9 (3.2 – 12.2)	0.96
Urea (mg/dL; RV: 17 – 43)	20 (15 – 24)	21 (16 – 26)	0.71
Na (mEq/L; RV: 135 – 145)	137 (136 – 138)	138 (136 – 139)	0.74
K (mEq/L; RV: 3.5 – 5)	4.7 (4.4 – 4.9)	4.7 (4.4 – 4.9)	0.91
Urine Osmolality (mOsm/kg; RV: > 600)	301 (296 – 305)	300 (296 – 301)	0.46
Plasma Osmolality (mOsm/kg; RV: 275-300)	736 (533 – 925)	529 (258 – 987)	0.57

RV: reference values. Data were compared using the Mann-Whitney *U* test.
For SI units, multiply by: 12.87 (FT4); 27.58 (cortisol); 0.22 (ACTH); 0.13 (IGF-1); 1 (IGFBP-3); 1 (prolactin); 0.05 (glucose); 6.94 (insulin); 0.35 (urea); 1 (Na), 1 (K); 1 (plasma and urine osmolality).

© 2021 Ferreira LL et al. *JAMA Network Open.*

eTable 4. Missing Values in Clinical and Developmental Characteristics

**Characteristics**	**Missing data (n) Mild or Moderate Microcephaly**	**Missing data (n) Severe Microcephaly**
Male, n (%)	0	0
Preterm, n (%)	5	0
Brain abnormalities, n (%)	0	0
Head circumference z-score, median (IQR)**	0	0
Congenital contractures – n (%)	0	0
Neonatal seizures – n (%)	0	0
Retinal anomalies – n (%)	2	0
Weight Z score – median (IQR) #	4	11
Length Z score – median (IQR) #	4	11
Head circumference Z score – median (IQR) #	4	13
Hearing loss – n (%)	4	7
Seizures – n (%)	0	0
Swallowing disorders – n(%)	1	1
Poor weight gain – n (%)	1	1
Sleep disorder – n (%)	0	0
Severe language delay – n (%)	0	1
Severe fine motor delay – n (%)	0	1
Severe gross motor delay – n (%)	0	1

© 2021 Ferreira LL et al. *JAMA Network Open.*

eTable 5. Missing Values in Hormonal Profile

Characteristics	Missing data (n) Mild or Moderate Microcephaly	Missing data (n) Severe Microcephaly
Age (months)	0	0
Free Thyroxine (ng/dL; RV: 0.54 – 1.43)	0	0
TSH (mUI/L; 0.5 – 6.6)	0	0
Cortisol (mcg/dL; VR: 5 - 18)	0	0
ACTH (pg/mL; 7- 30)	0	0
IGF-1 (ng/mL; RV: 49 - 270)	0	0
IGFBP-3 (mcg/mL; RV: 0.8 – 3.9)	0	0
Prolactin (ng/mL; RV: 2.6 - 25)	0	0
Glycaemia (mg/dL; RV: 70 - 100)	0	0
Insulin (mIU/L; RV: 2.5 - 25)	0	0
Urea (mg/dL; RV: 17 – 43)	8	12
Na (mEq/L; RV: 135 – 145)	1	4
K (mEq/L; RV: 3.5 – 5)	1	4
Urine Osmolality (mOsm/kg; RV: > 600)	20	25
Plasma Osmolality (mOsm/kg; RV: 275-300)	19	25

© 2021 Ferreira LL et al. *JAMA Network Open.*

eTable 6. Missing Values in Computed Tomography Scan Results

**Characteristics**	**Missing data (n) Mild or Moderate Microcephaly**	**Missing data (n) Severe Microcephaly**
Calcifications	0	0
Venticulomegaly	0	0
Hydrocephaly / Hydroanencephaly	0	0
Cerebral Atrophy	0	0
Lissencephaly / Pachygyria	0	0
Cerebellar Hypoplasia	0	0
Corpus Callosum's Agenesis or Hypoplasia	0	0

© 2021 Ferreira LL et al. *JAMA Network Open.*
